# Pathogenic bacteria profile and antimicrobial susceptibility patterns of ear infection at Bahir Dar Regional Health Research Laboratory Center, Ethiopia

**DOI:** 10.1186/s40064-016-2123-7

**Published:** 2016-04-16

**Authors:** Derese Hailu, Daniel Mekonnen, Awoke Derbie, Wondemagegn Mulu, Bayeh Abera

**Affiliations:** Bahir Dar Regional Health Research Laboratory Center, Bahir Dar, Ethiopia; Department of Medical Microbiology, Immunology and Parasitology, College of Medicine and Health Sciences, Bahir Dar University, P. O. Box 79, Bahir Dar, Ethiopia

**Keywords:** Ear infection, Pathogenic bacteria, Antimicrobial susceptibility, Ethiopia

## Abstract

Ear infection linked with frequent antibiotic prescription, hearing impairment, severe disability and death is a public health threat in developing countries. However, there is scarcity of documented data in the study area. Therefore, this study aimed at determining bacterial etiologic agents and their antimicrobial susceptibility patterns among patients of all age groups referred to Bahir Dar Regional Health Research Laboratory Center. Retrospective data recorded on culture and antimicrobial susceptibility profile were retrieved for analysis. Pus swabs from discharging ears collected and processed for aerobic bacteria culture and susceptibility testing. Of the total 368 pus swab samples processed, 296 (80.4 %) were culture positive. Of which, 289 (97.6 %) were bacteria and 7 (2.4 %) were yeast cells. The proportion of ear infection was higher in males (92.7 %) than females (65 %) (P = 0.014). The frequency of ear infection below 21 years of age was 65.2 %. The predominant isolate was *Pseudomonas aeruginosa* (29.7 %) followed by *Staphylococcus aureus* (26.3 %) and *Proteus* spp. (21.9 %). High level of antimicrobial resistance rates were observed for amoxicillin/clavulanic acid, ampicillin and penicillin whereas ciprofloxacin, ceftriaxone, chloramphenicol, cotrimoxazole, gentamicin and amikacin were found effective against the isolated bacteria. Aerobic bacterial otitis media linked with high levels of resistance against amoxicillin/clavulanic acid and ampicillin is major health problem in the study area. Moreover, considerable level of oxacillin resistant *S*. *aureus* suggests the diffusion of methicillin resistant *S*. *aureus* in the community. Therefore, treatment of otitis media in the study area needs to be guided by antibiotic susceptibility testing of isolates.

## Background

Otitis media is the inflammation of the middle ear cleft and the tympanum with otorrhoea lasting from 2 weeks to more than 3 months, with permanent perforation mainly caused by bacteria (Adoga et al. 2010; Mesfin and Muluken 2014). Ear infection may be acute or chronic purulent type (Wasihun and Zemene 2015). About 65–330 million people suffer from ear infection worldwide and 60 % of them had significant hearing loss (Woodfield and Dugdale 2008). Otitis media (OM) is a major health problem and occurs with a high incidence and prevalence in both developed and developing countries (Afolabi et al. 2012). The health and economic burden of ear infection is also severe especially in Africa and other developing countries where the disease prevalence is estimated as high as 11 % (WHO 2004). According to World Health Organization (WHO) report, Ethiopia belongs to among the high ear infection burden countries (WHO 2004).

Even though ear infection is primarily a disease of infants and young children, it can also affect adults (Farhan et al. 2011). The disease may begin in childhood or as a complication of untreated or inadequately treated acute suppurative otitis media or may be chronic from onset (Afolabi et al. 2012). The microorganisms may gain entry to the middle ear through a chronic perforation. Children tend to have higher predisposition to ear infection than adults because anatomy of the eustachian tube in children permits easier access of organism through the nasopharynx. Moreover, the incidence is higher in males than females (Afolabi et al. 2012; Seid et al. 2013).

The prevalence of otitis media varies from place to place. In the developed world like United States of America (USA) and Europe, it is declining because of awareness; but in developing countries, it is on the rise (Adoga et al. 2010). In developing countries untreated otitis media leads to purulent otitis often with perforation and further complications including recurrent acute otitis media, persistence of middle ear effusion which requires the insertion of drainage tube and often leads to hearing impairment, mastoiditis, meningitis, chronic otitis media, brain abscess and sepsis (Seid et al. 2013).

The etiology, frequency and antimicrobial resistance patterns of ear infection is different in different geographical area and climate conditions (Abera and Kibret 2011; Wasihun and Zemene 2015). According to reports of many studies, *Pseudomonas aeruginosa*, *Staphylococcus aureus*, *Proteus mirabilis*, *Klebsiella pneumoniae* and *Escherichia coli* are the common organisms isolated from cases of ear infection (Abera and Kibret 2011; Muluye et al. 2013; Seid et al. 2013).

Due to increased and irrational use of wide-spectrum antibiotics, development and spread of resistance in the bacterial isolates becomes a major global health threat (Agrawal et al. 2013; Wasihun and Zemene 2015). Increased antimicrobial resistant bacteria in ear infection can led to the development of complications such as meningitis and brain abscess (Abera and Kibret 2011). In addition, ear infection may leads to tumor in the middle ear, post aural swelling and aural sinus complications (Abera and Kibret 2011; Ahmad 2013).

The type, frequency and antimicrobial resistance pattern of bacterial etiologic agents of ear infection varies among populations due to variability in geography and local antimicrobial prescribing practices and prevalence of resistant bacterial strains. Due to the limited microbiology laboratory setup, physicians in the study area prescribe either of the following drugs: amoxicillin, amoxicillin/clavulanic acid, chloramphenicol, gentamicin or ciprofloxacin without the guidance of culture and antibiotic susceptibility tests to treat patients with presumptive of otitis media which could lead to emergence of drug resistance. Thus up-to-date information on microbial resistance needs to be available at national and local level to guide the rational use of the existing antimicrobials. Therefore, this study was carried out to determine bacterial etiologic agents of ear infections and their antimicrobial susceptibility profiles among patients who referred to Bahir Dar Regional Health Research Laboratory Center (BRHRLC).

## Methods

### Study design, period and area

A retrospective cross-sectional study was conducted based on review of records of 368 pus swab samples from discharging ears submitted for culture and antimicrobial susceptibility test to BRHRLC during the period of January 2013 to April 2015. It is the technical arm of Amhara Regional Health Bureau currently providing culture and susceptibility tests for different bacterial infections, and quality assurance services to Felegehiwot Referral Hospital, nearby health centers, private hospitals and clinics.

### Study participants and data collection

The study participants were all clinically suspected patients of otitis media that provided pus swab from discharging ears at BRHRLC during the study period. Age and sex profiles and results of bacterial isolates and drug susceptibility of patients who had ear infection were retrieved from the BRHRLC Microbiology Laboratory unit registration reports using a standard data recording format.

### Isolation and identification of bacteria

For the detection of pathogenic bacteria, all samples were collected by standard microbiological technique (Cheesbourgh 2006). Pus swab from discharging ears were collected using swab techniques by cotton–wool at the microbiology laboratory (Adoga et al. 2010). Ear discharge samples were inoculated on MacConkey agar, Blood agar, Mannitol Salt agar and Chocolate agar (Oxoid, UK). Blood and Chocolate agar plates were incubated in a candle jar at which can generate about 5 % CO_2_. All of the inoculated media were incubated at 37 °C for 18–24 h. Bacterial species were identified by standard microbiological methods manually (Cheesbourgh 2006).

### Antimicrobial susceptibility testing

Susceptibility testing was done on Mueller–Hinton agar using disk diffusion technique according to Kirby–Bauer Method (Bauer et al. 1966). The antimicrobial agents tested were: ampicillin (10 µg), amikacin (30 µg), ciprofloxacin (5 µg), cotrimoxazole (25 µg), piperacillin (100 µg), gentamicin (10 µg), tetracycline (30 µg), penicillin (10 IU), clindamycin (30 µg), ceftriaxone (30 µg), chloramphenicol (30 µg), ceftazidime (30 µg), erythromycin (15 µg), amoxicillin/clavulanic acid (30 µg) and oxacillin (30 µg) (Oxoid, England). The antibiotic susceptibility profiles were interpreted based on Clinical and Laboratory Standards Institute (CLSI 2014) guidelines.

### Quality control

A standard bacteriological procedure was followed to maintain correct laboratory test results. American Type Culture Collection (ATCC) standard reference strains *(S*. *aureus* ATCC 25923, *E*. *coli* ATCC 25922 and *P*. *aeruginosa* ATCC 27853) were used to control quality of culture and susceptibility testing.

### Statistical analysis

Data were entered and analyzed using SPSS statistical software package (IBM Corp. Released 2011. IBM SPSS Statistics for Windows, Version 20.0. Armonk, NY: IBM Corp.). Discrete variables were expressed as percentages. Chi square test was used to compare the proportion of bacterial isolates with patients’ age and sex and P value of <0.05 was considered statistical significant.

### Ethical considerations

Permission and ethical clearance was obtained from Amhara Regional Health Bureau Institutional Review Board (IRB) at Bahir Dar Regional Health Research Laboratory Center. Confidentiality was maintained. Result finding were communicated with ENT doctor to help the patients.

## Results

A total of 368 samples from clinically suspected patients of otitis media were tested and analyzed. Of them, 205 (55.7 %) were males and 163 (44.3 %) female patients. The mean age of patients was 13 year (range 1–72). Overall, 296 (80.4 %) of otitis media had microbial isolates. The proportion of ear infection was 190 (92.7 %) in males and 106 (65 %) in females (P = 0.014). Microbial isolates were detected in 102 (87.9 %) and 91 (78.4 %) patients from 11 to 20 and 1 to 10 years of age, respectively (Table 1).

**Table 1:** Distribution of ear infection in relation to age and sex of patients referred at Bahir Dar Regional Health Research Laboratory Center, 2015

Variables	Ear infection N (%)	Total N (%)	P value
Sex			
Male	190 (92.7)	205 (55.7)	0.014
Female	106 (65)	163 (44.3)	
Age (years)			
0–10	91 (78.4)	116 (31.5)	
11–20	102 (87.9)	116 (31.5)	0.07
21–30	51 (75)	68 (18.5)	
31–40	31 (75.6)	41 (11.1)	
41–50	17	17 (4.6)	
51–60	3	3 (0.8)	
≥61	1	7 (1.9)	
Total	296 (80.4)	368 (100)	

Of 296 isolates, 289 (97.6 %) were bacteria and 7 (2.4 %) were yeast cells. Gram negative bacteria were isolated most frequently in comparison to Gram positive (58.8 and 41.2 %, respectively). Nine (3.1 %) samples were observed with mixed bacterial growth (data not shown). The most frequent bacterial isolates were *P*. *aeruginosa* 88 (30.4 %) followed by *S*. *aureus* 78 (26.9 %) and *Proteus* spp. 65 (22.3 %) (Table 2).

**Table 2:** Frequency of microbial isolates (n = 296) of ear infection at Bahir Dar Regional Health Research Laboratory Center, 2015

Bacterial species	Rate of isolation	
	Frequency	Percentage
*P*. *aeruginosa*	88	30.4
*S*. *aureus*	78	26.9
*Proteus* spp.	65	22.3
CoNS	34	11.8
*K*. *pneumoniae*	10	3.5
*E*. *coli*	7	2.4
*S*. *pneumoniae*	7	2.4
*Total*	289	97.6
Yeast cells	7	2.4
Total	296	80.4

Key: *CoNS* Coagulase negative *Staphylococcus*

Overall, gram positive isolates revealed 4.2–46.2 % level of resistance pattern to the antimicrobials tested. Of 78 *S*. *aureus* isolates, 65.4, 42.6 and 34.6 % were resistant to penicillin, tetracycline and oxacillin, respectively. However, low level of resistance was found to cotrimoxazole, ciprofloxacin, erythromycin and chloramphenicol by Gram positive bacteria (Table 3). Gram negative isolates revealed 0.6–79 % level of resistance pattern to the antimicrobials tested. *P*. *aeruginosa*, *Proteus* spp., *E*. *coli and K*. *pneumoniae* were resistant to amoxicillin/clavulanic acid (66–90 %) and ampicillin (85.7–100 %). However, ceftazidime, piperacillin, ceftriaxone, gentamicin, amikacin, ciprofloxacin, chloramphenicol and cotrimoxazole were effective against most Gram negative bacterial isolates (Table 4).

**Table 3:** Antimicrobial resistance patterns of Gram positive bacterial isolates (n = 119) from pus swab taken from discharging ears of study participants, at Bahir Dar Regional Health Research Laboratory Center, 2015

Bacterial isolates		Resistance pattern of antimicrobial agents (R %)							
	#T	DA	OXA	TE	SXT	CAF	CIP	ERY	PEN
*S*. *aureus*	78	7.9	34.6	42.6	23.1	6.4	0.0	14.1	65.4
CoNS	34	2.9	5.9	17.6	5.9	0.0	0.0	0.0	11.8
*S*. *pneumoniae*	7	ND	ND	11.8	0.0	0.0	0.0	11.8	0.0
Total	119	6.7	24.4	25.3	16.8	4.2		12.6	46.2

Key: *CoNS* Coagulase negative *Staphylococcus*, *#T* total number of isolates tested against each antimicrobial agent, *R* *%* percent of isolates resistance to antimicrobial agent, *ND* not done, *DA* clindanycin, *OXA* oxacillin, *TE* tetracycline, *SXT* cotrimoxazole, *CAF* chloramphenicol, *CIP* ciprofloxacin, *ERY* erythromycin, *PEN* penicillin, *ND* not done

**Table 4:** Antimicrobial resistance profiles of bacterial isolates (n = 170) from pus swab samples taken from discharging ears study participants at Bahir Dar Regional Health Research Laboratory Center, 2015

Bacterial isolates		Resistance pattern of antimicrobial agents (R %)									
	#T	AMC	AMP	CIP	CRO	CAF	SXT	GEN	AK	PEP	CAZ
*P*. *aeruginosa*	88	89.0	90.9	8	23.0	6.8	0.0	10.2	4.5	10.2	31.8
*Proteus* spp.	65	66.0	87.8	4.6	27.7	0.0	0.0	23.1	0.0	12.3	21.5
*E*. *coli*	7	71.4	85.7	14.3	28.6	0.0	0.0	28.6	0.0	0.0	14
*K*. *pneumoniae*	10	89.9	100.0	1	20	10	10	20	0.0	10	20
Total	170	79	90	12	24.7	4.1	0.6	16.5	2.4	10.6	26.5

Key: *#T* number of isolates tested against each antimicrobial agent, *R* *%* percent of isolates resistance to antimicrobial agent, *AMP* ampecillin, *AMC* amoxicillin/clavulanic acid, *CRO* ceftriaxone, *AK* amikacin, *GEN* gentamycin, *CAZ* ceftazidime, *PEP* peperacillin

## Discussion

Ear infection is the most frequent disease for patients to visit clinicians and take antibiotics (Grevers 2010). In the study area, ear discharge sample is one of the frequently requested specimens for culture and antimicrobial susceptibility tests from clinical settings. In the same way, in this study, 80.4 % cases of discharging ear were found to be positive for bacteria. Likewise, with a slight variation several other authors in Ethiopia 91.7 % (Abera and Kibret 2011), 89.5 % (Muluye et al. 2013), 89.4 % (Seid et al. 2013) and 98.2 % (Wasihun and Zemene 2015) were reported to be a similar result. This indicates that otitis media is a common health problem of the society.

In the present study, the proportion of ear infection was significantly higher in males compared to females (P = 0.014). This finding corroborates to results from Ethiopia (Muluye et al. 2013) and Nigeria (Egbe et al. 2010). In contrast, in reports of Hassan and Adeyemi (2007) females were more affected by ear infection than males. On the other hand, other studies (Abera and Kibret 2011; Seid et al. 2013; Wasihun and Zemene 2015), reported the absence of significant difference on the incidence of ear infections between males and females.

In this study, the highest proportion of otitis media was found among patients from 1 to 20 years of age which agrees with reports from Ethiopia (Muluye et al. 2013; Seid et al. 2013) and other countries (Ahmad 2013; Iseh and Adegbite 2004). Higher frequency of ear infection among young age groups might be due to the short, broad and straight nature of the Eustachian tube, lack of hygiene, lower immune status, frequent exposure to upper respiratory tract infections and malnutrition (Ahmad 2013; Muluye et al. 2013; Seid et al. 2013; Wasihun and Zemene 2015).

In the present study, gram-negative bacteria were the dominant isolates (58.8 %) of ear infection compared to gram-positive bacteria which is in agreement to earlier studies in Dessie where 74.2 % (Abera and Kibret 2011), Gonder 56.4 % (Muluye et al. 2013), Hawassa 79.5 % (Mesfin and Muluken 2014), Mekelle 56 % (Wasihun and Zemene 2015) and Nigeria 75 % (Iseh and Adegbite 2004) of isolates were shown to be Gram positive though the proportion varies. In this study single bacterial infection of the ear was seen in 96.9 % of patients. This observation was supported by other researchers elsewhere (Abera and Kibret 2011; Seid et al. 2013; Abdelraouf et al. 2014).

In this study, *P*. *aeruginosa* (30.4 %) followed by *S*. *aureus* (26.9 %) and *Proteus* spp. (22.3 %) were the leading isolates. This trend is similar to reports of other researchers (Aslam et al. 2004; Iseh and Adegbite 2004; Weckwerth et al. 2009; Fatima et al.^2013^; Raghvendra et al. 2013). In contrast to our results, *Proteus* spp., followed by *S*. *aureus* and *Pseudomonas* spp. were the predominant isolates as found by others (Muleta et al. 2004; Abera and Kibret 2011; Muluye et al. 2013; Seid et al. 2013; Denboba et al. 2016). Although variation in climate and geography could be the possible reasons for the difference in distribution of the leading isolates, further nationwide study is important. The most common isolation rate of *P*. *aeruginosa* in our study could be related to the ability of *P*. *aeruginosa* to survive in competition with other organisms and resist to antibiotics. Moreover, *P*. *aeruginosa* uses its pili to attach to the necrotic or diseased epithelium of the middle ear. Once attached, the organism produces enzymes like proteases to elude the normal defense mechanism of body required for fighting infections (Seid et al. 2013).

The prevalence of *K*. *pneumoniae* and *E*. *coli* in this study was 3.4 and 2.4 %, respectively. Likewise, 11.1 and 3.7 % of *K*. *pneumoniae* and *E*. *coli* was, respectively reported in Ethiopia (Wasihun and Zemene 2015). Moreover, a study in India (Prakash et al. 2013) reported 8 and 4 % of *K*. *pneumoniae* and *E*. *coli*, respectively though the proportion varies. Isolation of fecal bacteria like *K*. *pneumoniae* and *E*. *coli* might indicate that individuals were at risk of infection due to poor hygiene conditions.

Although bacteria are the most frequent causes of ear infections, fungi flourish well in moist discharging ear. However, the proportion of ear infection due to yeast cells was not studied before in Ethiopia. Thus, this study presents the isolation rate of yeast cells in ear infection. In this study yeast cells isolated in 2.4 % of the cases which is consistent with a study in Pakistan (2 %) (Fatima et al. 2013) and United Arab Emirates (2.8 %) (Suman and Heba 2014).

In this study *P*. *aeruginosa* showed high level of resistance (89–90.5 %) to amoxicillin/clavulanic acid and ampicillin. This was consistent with a report in Dessie (Abera and Kibret 2011; Seid et al. 2013), Gondar (Muluye et al. 2013), where 86–91.8 and 60–67 % resistance levels of ampicillin and amoxicillin/clavulanic acid, respectively were noticed. However, *P*. *aeruginosa* was highly sensitive to ciprofloxacin, chloramphenicol, gentamicin and amikacin. This was consistent with reports from Ethiopia (Abera and Kibret 2011; Wasihun and Zemene 2015).

In the present study, 65.4 and 34.6 % of *S*. *aureus* isolates were resistant to penicillin and oxacillin, respectively. This was supported by findings of others (Hwang et al. 2002; Abera and Kibret 2011; Ahmad 2013; Muluye et al. 2013; Seid et al. 2013). This suggests the diffusion of *S*. *aureus* strains resistant to all β-lactam antibiotics and methicillin in the community which gives an alert for further large scale study on the prevalence and susceptibility patterns of community acquired Methicillin resistant *S*. *aureus* (CoMRSA) otitis media in Ethiopia.

On the other hand, *S*. *aureus* exhibited low levels of resistance (0–23.1 %) to ciprofloxacin, chloramphenicol, clindamycin, erythromycin and cotrimoxazole. These were consistent with the results of other studies (Muleta et al. 2004; Abera and Kibret 2011; Seid et al. 2013; Muluye et al. 2013; Wasihun and Zemene 2015) for ciprofloxacin and erythromycin.

In the present study, *Proteus* spp. showed high level of resistance (88.7 %) to ampicillin and moderate resistance (66 %) to amoxicillin/clavulanic acid. However, *Proteus* spp. exhibited low levels of resistance (5–28 %) to ciprofloxacin, piperacillin, ceftazidime, gentamicin and ceftriaxone. These were consistent with other findings (Ferede et al. 2001; Abera and Kibret 2011; Seid et al. 2013; Muluye et al. 2013; Wasihun and Zemene 2015).

The presence of high levels of resistance to amoxicillin/clavulanic acid, ampicillin, penicillin, and oxacillin in the three leading pathogenic bacteria associated with otitis media in this study might be due to lack of up to-date knowledge on antimicrobial resistance among physicians and nurses, unavailability of local antibiogram data, misuse of antibiotics, and self-prescription by patients and negligence on patient part.

In conclusion, bacterial ear infection is a major health problem in the study area. *P*. *aeruginosa*, *S*. *aureus* and *Proteus* spp. were the dominant isolates. Most of the isolates were linked with high levels of resistance against amoxicillin/clavulanic acid and ampicillin. Moreover, considerable level of oxacillin resistant *S*. *aureus* suggests the diffusion of methicillin resistant *S*. *aureus* in the community. However, cotrimoxazole, ciprofloxacin, chloramphenicol and gentamicin were effective against most of the bacterial isolates. Therefore, treatment of otitis media in the study area needs to be guided by antibiotic susceptibility testing of isolates. Moreover, prevalence and susceptibility patterns of CoMRSA and extended spectrum β-lactamase (ESBL) production for the commonly isolates of Gram negative bacteria should be further studied.

## Limitation

Because of the limited patient details entered in the register it was impossible to provide clinical and previous therapeutic details. No data was mentioned in the register about ESBL production.
